# Navigation Experience in Latin-American Spine Surgeons: A Survey

**DOI:** 10.7759/cureus.74723

**Published:** 2024-11-29

**Authors:** Felipe Aguilar-Chávez, Fernando González-González, Maria E Martinez-Tapia, Carlos A Arellanes-Chavez

**Affiliations:** 1 CISNE Spine Academy, Star Medica Hospital, Autonomous University of Chihuahua, Chihuahua, MEX; 2 Department of Epidemiology, Health Services of the State of Chihuahua, Chihuahua, MEX

**Keywords:** internet-based opinion survey, latin america, spinal navigation, spine, spine surgery

## Abstract

Study design: This is a cross-sectional survey.

Objectives: This study aimed to evaluate the availability and knowledge of navigation technologies for educational purposes and patient management in spine surgeons in Latin America.

Methods: A cross-sectional study was conducted among Latin American Spine Association members using a comprehensive 16-question survey to evaluate their knowledge and practices regarding navigation in spinal surgery. The questionnaire was reviewed and authorized by the AO Spine Latin America (LATAM) Degenerative & Deformity study group and distributed starting on January 29 and closed on February 28, 2024.

Results: A total of 123 surveys were recorded; 95% were male gender, and 42% were neurosurgeons/orthopedists with specific training in spine surgery. Mexico led the response rate with 55 (45%) and then Brazil and Argentina with 13% and 9%, respectively; 54% are not involved in a spine surgery training program; 80 surgeons belong to AO Spine membership; and 35 of them have over 20 years of experience, with most respondents performing between 0 and 100 surgeries per year and degenerative pathology being the most common. Almost 90% of the respondents either use or are willing to use navigation technology, 100% express interest in attending a course on the subject, 80% cited improved accuracy in screw placement as a perceived advantage, and the disadvantage of high costs was the most frequently selected (85%).

Conclusions: Despite the benefits demonstrated by navigation technology, many spine surgeons in Latin America still lack access to this valuable resource. Even with their keen interest and clear understanding of its advantages and benefits, it is necessary to develop training programs and affordable navigation systems to improve spine surgery worldwide and ensure accessible care for all.

## Introduction

Navigation in spinal surgery is a relatively new technology, revolutionizing treatment in this field. The purpose is to have a real-time three-dimensional image to facilitate procedures [1], in addition to specific advantages compared to other techniques, such as reduced radiation exposure, especially for the surgical team, greater accuracy in the placement of transpedicular screws, the convenience of not using protective vests during surgery, and improved operating room and surgical field space due to the absence of the C-arm [2].

Another aspect to take into account pertains to the medicolegal consequences associated with the improper positioning of the pedicle and lateral mass screws during spinal surgery [3], particularly in the United States. Neurosurgery is notably the medical specialty most commonly entangled in legal disputes and anxiety over potential domestic and international litigation, with spine surgery occupying a prominent role in this context [4].

In previous years, Latin American spine surgeons have reported limited access to various technologies for performing minimally invasive surgeries, even in private practice. However, it is important to highlight the benefits of these methods and the understanding of this knowledge in this region of the world today [5].

Similarly, in 2013, Hartl et al. surveyed with the same objective of clarifying the understanding of navigation technology available at that time [6]. However, a decade has elapsed since then, and this technology has undergone substantial advancements, marked by the introduction of new equipment and a seemingly enhanced knowledge base among surgeons [7].

This study aimed to evaluate the availability of spine navigation technologies for educational purposes, patient management in spine surgeons in Latin America, and the knowledge about it.

## Materials and methods

In this cross-sectional study, we designed an extensive 16-question survey (see Appendix) to assess general knowledge about the application of navigation in spinal surgery in multiple Latin American Association members like the Association of the Study of Internal Fixation Spine Division in Latin America (English acronym: AO Spine LATAM (Latinoamerica)), the Latin American Federation of Neurosurgery Societies (Spanish acronym: FLANC (Federación Latinoamericana de Sociedades de Neurocirugía)), the Mexican Association of Spine Surgeons (Spanish acronym: AMCICO (Asociación Mexicana de Cirujanos de Columna)), the Spine Webinar Mexico and the Mexican Society of Neurological Surgery (Spanish acronym: SMCN (La Sociedad Mexicana de Cirugía Neurológica A.C.)). The questionnaire covers aspects such as gender, specialization, and geographic location, including country and hospital. We also investigate the availability of resources in respondents’ hospitals for the execution of navigation-assisted surgeries, as well as the annual number of surgeries performed and the portion of them that utilize navigation technology or other techniques.

Furthermore, we seek to comprehend the reasons underlying the choice to employ navigation or opt against it in these surgical procedures. We also aim to determine if respondents have taken part in spinal surgery navigation courses and if they are AO Spine members. We also explore whether their hospitals offer specialized training programs in spinal surgery.

It is worth noting that almost all questions provide predefined response options, except for the inquiry regarding respondents' hospital names and the increased time in minutes in navigation surgery, which remains an open-response field in the perceived disadvantages question.

The questionnaire was developed by three senior neurosurgeons with experience in spinal surgery (CA, JF, and AP) in active collaboration with two fellows in spinal surgery (FA and FG) during August and September 2023 and then was analyzed by the AO Spine LATAM Degenerative & Deformity study group and authorized the distribution to all the actual members of AO Spine LATAM countries by e-mail from the AO Spine LATAM membership database since January 29, 2024, with a reminder on February 23. Then, we sent the invitation by WhatsApp to FLANC, AMCICO, Spine Webinar Mexico, and SMCN members. The survey was closed on February 28, 2024.

Finally, we sent 9,360 emails, 1,633 in Portuguese and 7,727 in Spanish. However, the exact number of members to whom it was sent is not available, because when sending the invitations, there were technical errors that made this measurement impossible. We shared the survey by WhatsApp invitation to 201 FLANC members, 601 Spine Webinar members, 410 AMCICO members, and 311 SMCN members, although many members belong to various groups. Therefore, the exact total number of members is not available.

Statistical analysis

The data were organized and analyzed in Minitab software (version 21.0; Minitab LLC, State College, PA, USA). Categorical variables were described using absolute and relative frequencies and quantitative variables, measures of central tendency, and dispersion were used. To compare the advantages and disadvantages of the three specialties, Chi^2^ with a value of p = 0.05 was used to establish statistical significance.

## Results

Characteristics of the study population

Responses were obtained from 15 Latin American countries. The majority of responses belonged to Mexico with a total of 55 responses (44.72%), followed by Brazil with 16 responses (13.01%) and then Argentina and Colombia with 11 each (17.88%) (Table 1).

**Table 1:** Participant distribution by country of origin across Latin America.

Country	Frequency	Percentage
Argentina	11	8.94%
Brazil	16	13.01%
Chile	8	6.50%
Colombia	11	8.94%
Costa Rica	1	0.81%
Ecuador	2	1.63%
El Salvador	2	1.63%
Guatemala	3	2.44%
México	55	44.72%
Panamá	2	1.63%
Paraguay	2	1.63%
Perú	4	3.25%
Dominican Republic	1	0.81%
Uruguay	1	0.81%
Venezuela	4	3.25%

**Figure 1:**
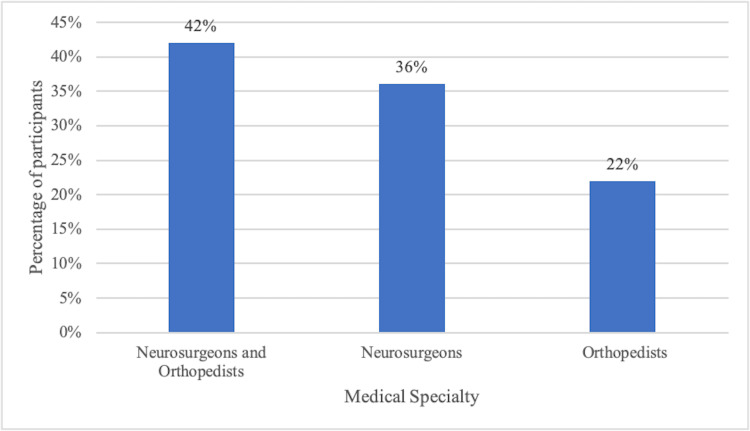
Distribution of participants according to medical specialty.

A total of 123 surveys were recorded, of which six (5%) were females and 117 (95%) were males. The majority confessed not being part of any spine surgery training program, with 66 responses (53.66%); eight (6.5%) were students and 49 (39.84%) were adjunct professors in the academic program at their hospital. There was a majority of specialists (neurosurgeons and orthopedists) with training in spine surgery, totaling 52 (42%). The rest were neurosurgeons, with 44 (36%) and orthopedists with 27 (22%) (Figure 1).

Characteristics of surgical practice

**Figure 2:**
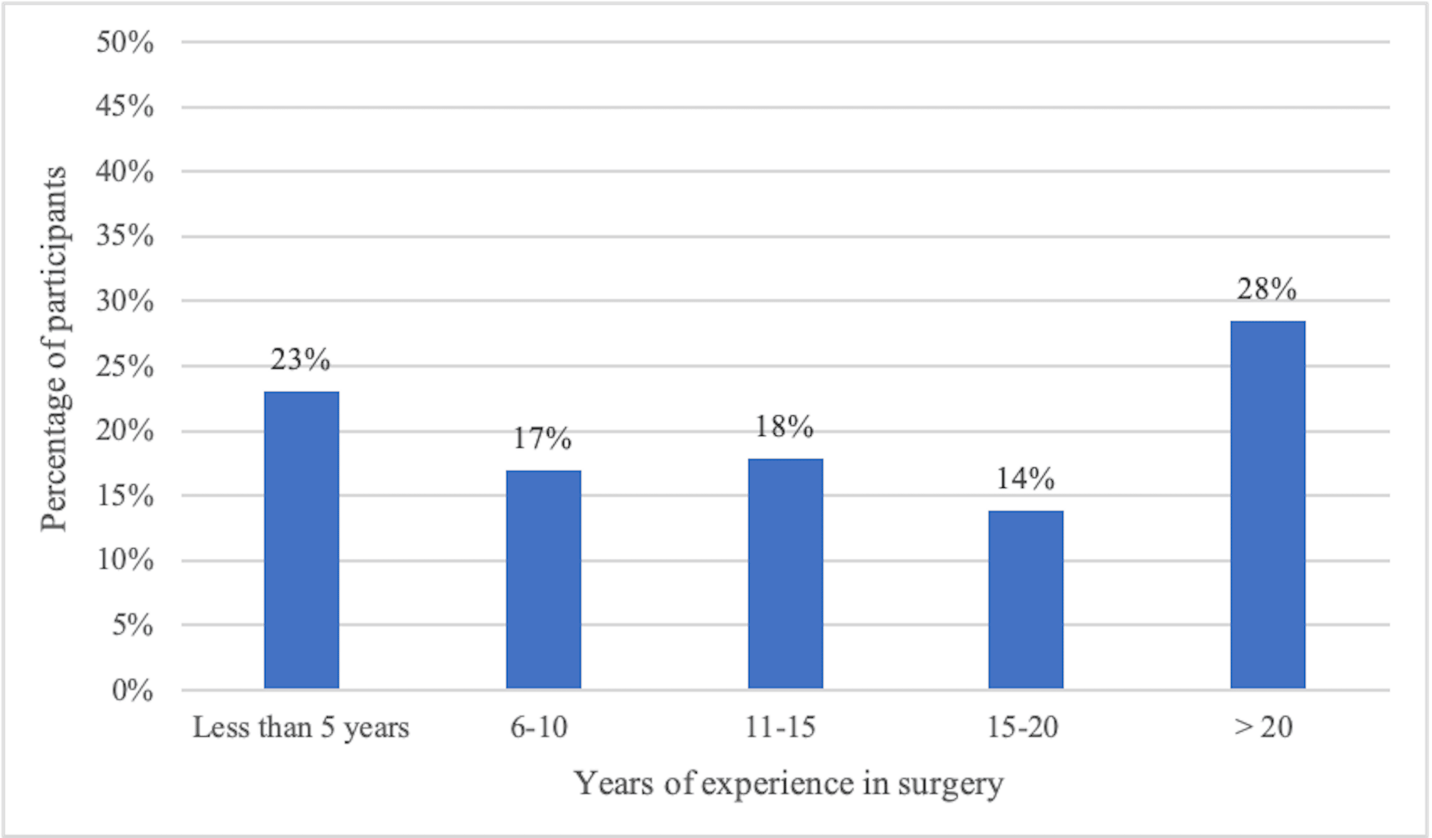
Distribution of participants according to years of experience in surgery.

Of the 123 respondents, 80 (65.04%) are AO Spine members. Regarding experience, 35 (28.46%) have over 20 years in spine surgery, 17 (13.82%) have 15-20 years, 21 have 11-15 years, 21 have six to 10 years, and 28 (22.76%) have less than five years (Figure 2).

Annual surgical volumes show 35 surgeons performing 0-50 surgeries (28.46%), 39 performing 51-100 (31.71%), 19 performing 101-150 (15.45%), 21 performing 151-200 (17.07%), and nine (7.32%) performing over 200. Specifically, one surgeon reported 201, four reported 250, one reported 300, one reported 360, and two reported 400 surgeries annually.

**Figure 3:**
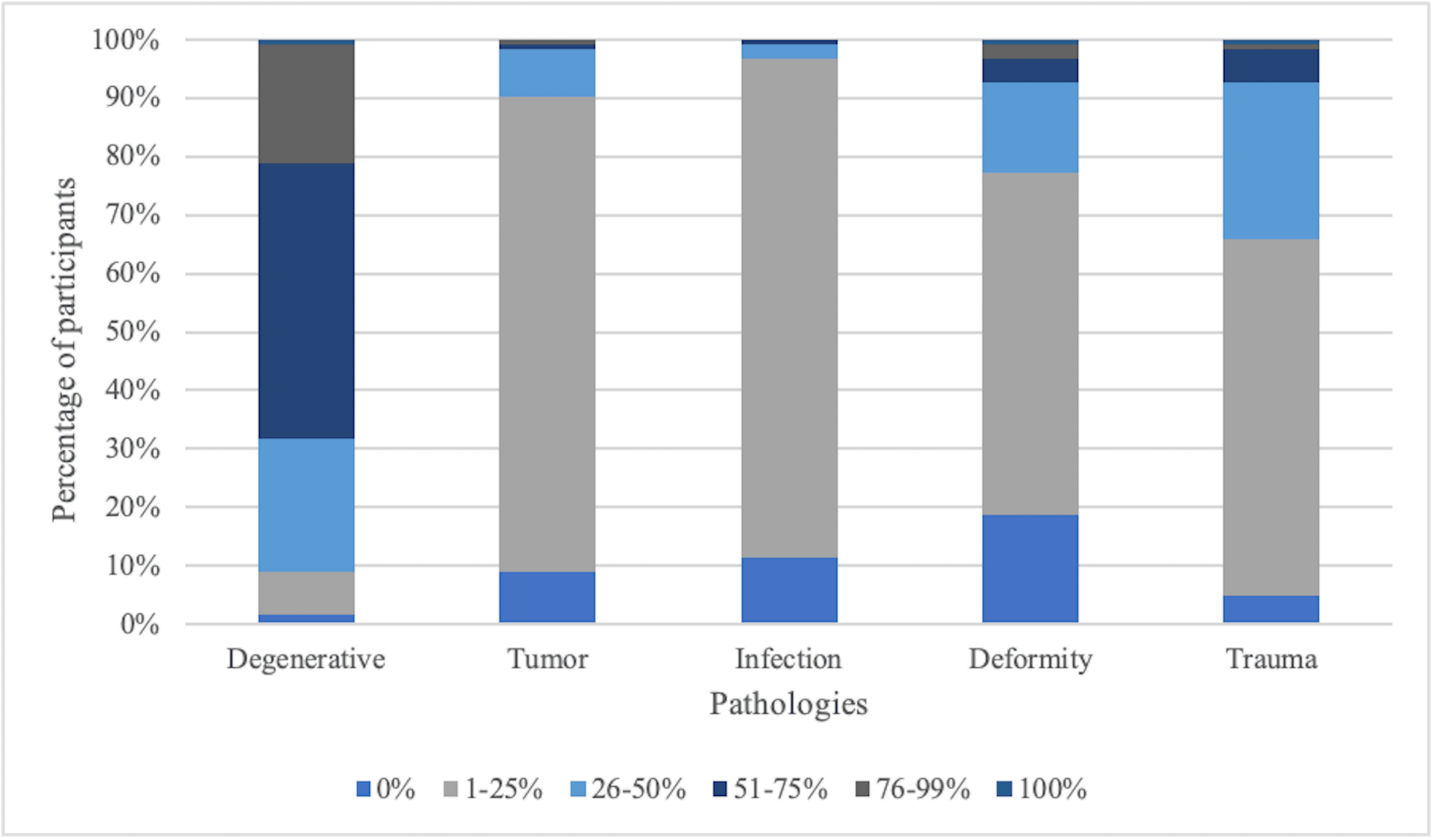
Percentage distribution of spinal pathologies treated routinely in the clinical practice.

Surgeons were asked about the percentage of their patients presenting with degenerative diseases, tumors, infections, deformities, or trauma. For degenerative diseases, most surgeons (47.15%) treat 51-75% of patients with this condition, while only one treats it exclusively. Tumor cases are less common, with 81.30% treating 1-25% of patients, and no surgeon treating tumors exclusively. For spine infections, 85.37% of surgeons report 1-25% of their caseload, and none treat more than 75%. Deformities are seen in 1-25% of cases for 58.54% of surgeons, while only one surgeon treats this exclusively. Spinal trauma is reported by 60.98% of surgeons in 1-25% of their patients, with just one treating trauma exclusively (Figure 3).

In the comparison of the pathologies between the specialties, a significant difference was observed in the performance of degenerative surgeries (X^2^ = 25.8, p = 0.0039), while orthopedic/neurosurgeons with additional training in spinal surgery have a higher proportion in this category compared to neurosurgeons and orthopedic surgeons. There is also a significant difference in the performance of surgeries for tumors (X^2 ^= 18.3, p = 0.019), with neurosurgeons performing the majority of surgeries for tumors, followed by orthopedic/neurosurgeons with additional training in spinal surgery. No significant differences were found between the specialties in the performance of surgeries for infection (X^2 ^= 6.92, p = 0.328), deformity (X^2 ^= 14.4, p = 0.1545), and trauma (X^2 ^= 13.2, p = 0.2087).

Computer-assisted navigation

**Figure 4:**
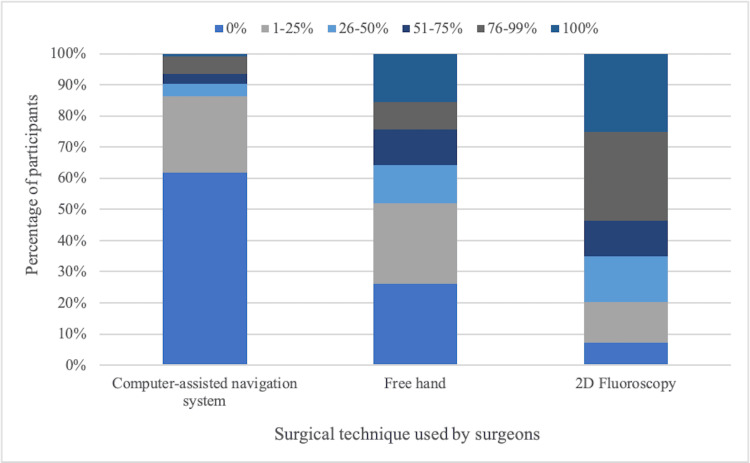
Percentage distribution of surgical techniques used by surgeons.

We also inquired about the most frequently used techniques for spinal surgery: computer-assisted navigation, hands-free, or 2D fluoroscopy. Regarding the navigation system, 76 surgeons (61.79%) reported using it in 0% of their surgeries, while 30 (24.39%) use it for 1-25%, five (4.07%) for 36-50%, four (3.25%) for 51-75%, seven (5.69%) for 76-99%, and only one surgeon for 100% of their surgeries. For hands-free surgery, 32 surgeons (26.02%) reported using it in 0% of cases, 32 (26.02%) for 1-25%, 15 (12.20%) for 26-50%, 14 (11.38%) for 51-75%, 11 (8.94%) for 76-99%, and 19 (15.45%) for 100% of surgeries. In terms of 2D fluoroscopy, nine surgeons (7.32%) use it in 0% of cases, 16 (13.01%) for 1-25%, 18 (14.63%) for 26-50%, 14 (11.38%) for 51-75%, 35 (28.46%) for 76-99%, and 31 (25.20%) for 100% of surgeries (Figure 4).

We surveyed surgeons to assess their use of fusion in spine surgeries and their knowledge of navigation technology. Of the 123 respondents, 16 (13.01%) reported that 0-25% of their surgeries involve fusion, 48 (39.02%) said 26-50%, 45 (36.59%) indicated 51-75%, and 14 (11.38%) noted 76-100%.

Regarding navigation technology, 38 surgeons (30.89%) use it, while 10 (8.13%) have the equipment but do not use it. Seventy-two surgeons (58.54%) lack the system but would use it if available, and three (2.44%) would not use it, even if they had access. In terms of training, 58 surgeons (47.15%) have not taken any courses but are interested, while 65 (52.85%) have received training.

When asked about the benefits of computer-assisted navigation, 98 surgeons (80%) believe it improves precision, 44 (36%) think it reduces surgical time, 89 (72%) agree it decreases radiation exposure, and 57 (46%) see its value in enhancing preoperative and intraoperative planning. In addition, 83 surgeons (67%) believe it aids in complex surgeries, 56 (45%) think it is helpful in revision surgeries, and 40 (33%) see it as useful in medico-legal matters. Finally, 53 surgeons (43%) find it beneficial for minimally invasive procedures, and 77 (63%) appreciate its role in controlling the position of transpedicular screws (Table 2).

**Table 2:** Advantages of computer-assisted navigation in spine surgery.

Advantages of computer-assisted navigation in spine surgery	Frequency	Percentage
Improves accuracy (level and position of the screw)	98	80%
Decreases surgical time	44	36%
Decreases radiation exposure for the surgical team	89	72%
Ameliorates preoperative and intraoperative planning	57	46%
Assists with complex (>5 levels, cervical)	83	67%
Assists with revision surgeries	56	45%
Provide support in the case of medical-legal issues	40	33%
Offers assistance in minimally invasive techniques	53	43%
Intraoperative image control of screw position	77	63%

Regarding the disadvantages of computer-assisted navigation in spine surgery, the most reported issue is its cost, cited by 104 surgeons (84.55%), followed by the lack of availability (60 surgeons, 48.78%). Other concerns include the surgeons' lack of knowledge on how to use the system (20 surgeons, 16.26%), medical insurance reluctance to approve its use (50 surgeons, 40.65%), and system incompatibility (27 surgeons, 21.95%). In addition, some surgeons mentioned that it increases surgery time (26 surgeons, 21.13%) or that it results in more radiation exposure (six surgeons, 4.87%). Only a small percentage felt it was unnecessary (2 surgeons, 1.62%), and one surgeon (0.81%) listed other unspecified disadvantages (Table 3).

**Table 3:** Disadvantages of computer-assisted navigation in spine surgery.

Disadvantages of computer-assisted navigation in spine surgery	Frequency	Percentage
Not available	60	48.78%
It is expensive	104	84.55%
I do not know how to use it	20	16.26%
More radiation exposure	6	4.87%
It is not necessary	2	1.62%
Medical insurance is reluctant to approve	50	40.65%
Incompatibility between systems available	27	21.95%
Increase the time of surgery	26	21.13%
Others	1	0.81%

The results regarding the increase in surgery time due to the use of computer-assisted navigation show that most surgeons reported varying increases in surgical duration. Specifically, 8% noted a 20-minute increase, 28% observed a 30-minute increase, 8% experienced a 45-minute delay, 4% saw a 50-minute increase, 32% reported a 60-minute increase, 12% experienced a 90-minute increase, and 8% saw a 120-minute delay. Overall, 25 surgeons provided responses on this issue.

Regarding the advantages, there is no significant difference among the specialties in terms of improving precision (X^2 ^= 5.02, p = 0.08). However, a higher proportion of neurosurgeons and orthopedic/neurosurgeons with additional training in spinal surgery perceive improvement in precision compared to orthopedic surgeons was identified. No significant difference was observed among the specialties in reducing surgical time (X^2 ^= 0.78, p = 0.67), reducing radiation exposure (X^2 ^= 1.52, p = 0.46), enhancing preoperative and intraoperative planning (X^2 ^= 0.27, p = 0.87), and assisting in complex surgeries (X^2^ = 2.25, p = 0.32).

**Figure 5:**
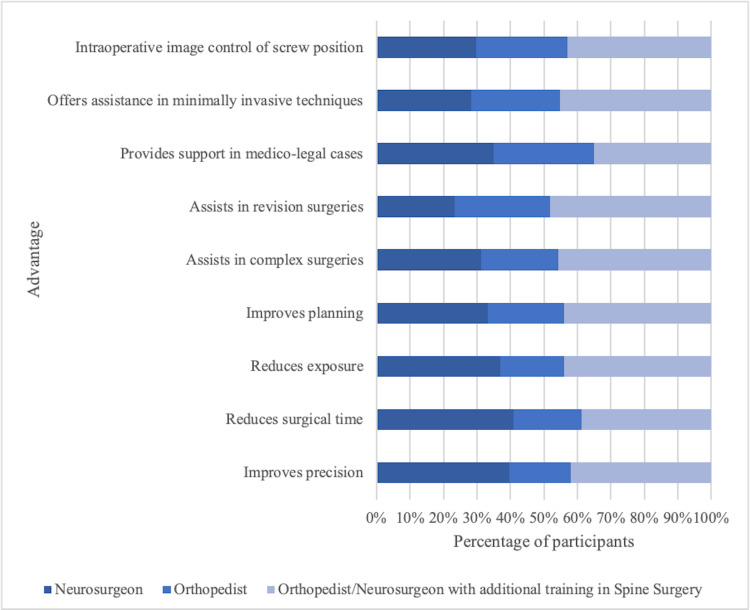
Advantages of different specialties of computer-assisted navigation in spine surgery.

A significant difference was observed among the specialties in assisting in revision surgeries (X^2 ^= 7.44, p = 0.02) since a higher proportion of neurosurgeons and orthopedic/neurosurgeons with additional training in spinal surgery think that this technology assists in these cases compared to orthopedic surgeons. No significant difference was found among the specialties in providing support in the case of medicolegal issues (X^2 ^= 2.50, p = 0.28), offering assistance in minimally invasive techniques (X^2 ^= 2.49, p = 0.28), and the intraoperative image control of the screw position (X^2 ^= 467, p = 0.09) (Figure 5).

**Figure 6:**
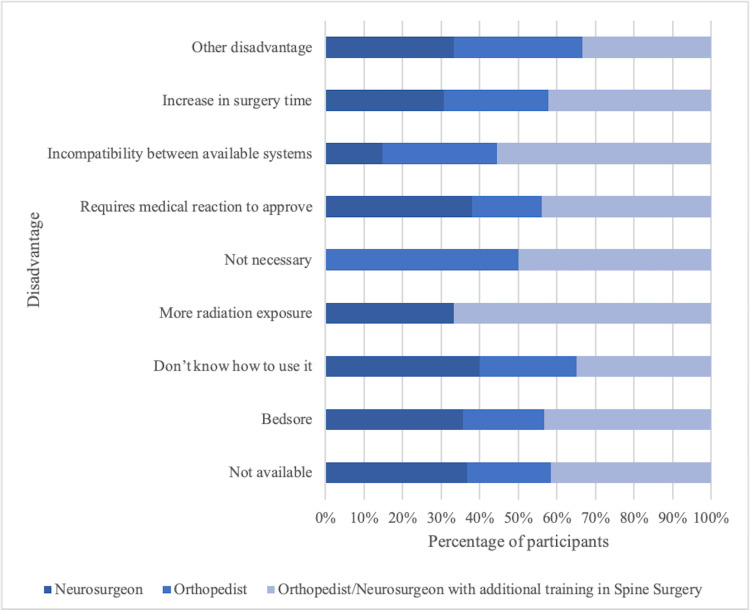
Disadvantages of different specialties of computer-assisted navigation in spine surgery.

Regarding the disadvantages, there is no significant difference among the specialties regarding unavailability (X^2 ^= 0.04, p = 0.97), in terms of perceived costliness (X^2 ^= 0.35, p = 0.83), knowing how to use the equipment (X^2 ^= 0.51, p = 0.77), the requirement of the equipment (X^2 ^= 1.48, p = 0.47), medical insurance reluctance to approve (X^2 ^= 0.77, p = 0.67), and increase in surgery time (X^2 ^= 0.60, p = 0.74). Just a significant difference was observed among the specialties concerning the incompatibility between available systems (X^2 ^= 6.61, p = 0.03), as the orthopedic surgeons and orthopedic/neurosurgeons with additional training in spinal surgery perceive higher incompatibility compared to neurosurgeons (Figure 6).

## Discussion

This study primarily evaluates the availability and knowledge within the Latin American spine surgeon community regarding computer-assisted navigation. In addition, we assessed various characteristics of the respondents and issues within their practice. Our findings revealed an overwhelming majority of 95% male participants, which aligns with trends in spinal surgery where there is a disparity in the number of male and female surgeons both in the field and in academic aspects such as conference presentations. This is despite other specialties showing different growth patterns [8-10].

It is well-known that in some Latin American countries, the specialty of spinal surgery is not as well-established with proper monitoring or certification as regularly done with other specialties or subspecialties [11].

Orthopedic surgeons and neurosurgeons can perform specific spinal procedures, and while the number varies by specialty, there has been a slight increase, especially among orthopedists surpassing the growth seen in neurosurgery at least in the USA [12].

We found that the majority of respondents have received training in spinal surgery (42%), and among them, a greater number of general neurosurgeons (36%) exceed general orthopedists (22%).

Degenerative pathology is the most commonly treated condition, which aligns with the literature [13-14]. However, nearly 80% of surgeons only perform a fusion procedure in 25% to 75% of their cases, suggesting that half of the patients they operate on may not present instability or reasons for fusion. This, however, can vary depending on the surgeon's criteria [15].

Sixty percent of respondents claim to have the necessary equipment for computer-assisted navigation procedures, and 30% admit to not having the equipment but would use it if available, which demonstrates an improvement in the percentage of availability of this technology compared to previous studies [6]. This is encouraging, as with unlimited resources, almost 100% would be willing to use this technology. In addition, 100% of the respondents admit to either having attended a course on this technology or intending to attend one to familiarize themselves with the equipment, which demonstrates a keen interest in navigation systems.

This also aligns with previous surveys indicating limited experience and exposure, at least in Latin America [6].

Another finding is that 47% are involved in some form of spine surgeon training program as either teachers or students; something that has been proven previously is that computer-assisted navigation is a more useful tool for training students than other conventional methods [1].

What is interesting about this study are the perceived advantages and disadvantages presented by respondents regarding this technology, which have been studied in multiple studies [16-20].

Among the perceived advantages, 80% believe that it improves screw placement effectiveness and locating the appropriate level for surgery, which substantially aligns with literature findings [21].

Another highly studied and demonstrated advantage of this technology is the significant reduction in surgical staff radiation exposure [22], which 72% of respondents believe is a considerable advantage when choosing this technology.

Regarding complex spinal surgery, like conditions in the cervical region and more than five levels where computer-assisted navigation is widely useful with screw insertion accuracy and reducing of bleeding [16-17], 67% of the respondents believe that this technology offers an advantage in this type of scenario, making it the third most mentioned advantage after screw placement effectiveness and reduction in surgical staff radiation exposure.

One other perceived advantage with a high voting percentage of 63% is the ability to monitor screw placement intraoperatively, a benefit also supported by the literature [21].

Spinal surgery is an area heavily impacted by medicolegal issues, with one reason being the poor placement of pedicle screws or the material used, which can cause multiple complications [4,23].

Despite the literature demonstrating that this technology improves surgical outcomes and serves as a tool to reduce complications and protect surgeons from radiation exposure and potential legal claims, there are perceived disadvantages to this technology.

The most notable disadvantage, with 85% of responses, is the high cost of the equipment and materials required for these procedures. Similarly, 40% agreed that insurance companies in these regions are reluctant to include navigation payments in their services due to the high costs associated with this technology. However, this technology reduces reoperation rates due to symptomatic screw misplacement and proves cost-effective in high-volume centers with better patient outcomes, meaning that the high acquisition and maintenance costs of these technologies are economically justified [24].

Another disadvantage pointed out by our participants is the lack of availability of this technology in their hospital centers, a common occurrence in Latin America for all types of necessary patient care supplies [5].

Spinal navigation technology offers clear advantages, such as improved accuracy in screw placement, reduced radiation exposure for surgical staff, and enhanced safety in complex procedures, aligning with the literature findings we mentioned. However, significant barriers persist, including the high costs of equipment and materials, limited availability in hospital settings, and reluctance from insurance providers to cover these expenses. Despite these challenges, the technology’s ability to lower reoperation rates and improve patient outcomes makes it a valuable investment, especially in high-volume centers where its cost-effectiveness is more apparent. Addressing these limitations is crucial for broader implementation, particularly in regions like Latin America, where accessibility remains a critical issue.

Limitations

In this study, we encountered challenges in calculating the response rate due to inherent limitations in participant identification and outreach methods. Specifically, the exact number of individuals to whom invitations were sent via email to AO Spine members remained undefined owing to technical difficulties. Furthermore, while invitations were disseminated through WhatsApp groups affiliated with various associations, the presence of overlapping memberships hindered precise quantification. Consequently, the observed 123 responses represent a notably low figure, approximately 2% of the total invitations presumed to have been dispatched. This highlights a concerning lack of engagement among spinal surgeons in research endeavors of this nature. To address these limitations, we advocate for the implementation of more personalized and targeted surveys tailored to this demographic. In addition, expanding outreach efforts to include broader associations within the Latin American spinal surgeon community is recommended to garner a more representative response.

## Conclusions

Updating the Latin American spine surgeon communities about this technology is essential; however, there is a lot of interest on the part of spine surgeons to learn and use this technology as computer-assisted navigation has been on the rise over the past two decades and is here to stay and continue growing. It is something that will improve surgical outcomes and significantly reduce the rate of complications and excessive costs due to readmissions and revision surgeries in patients. 

To address this gap, it is imperative to develop more training programs and specialized courses aimed at equipping surgeons with the necessary skills to utilize navigation systems effectively. These educational initiatives should be tailored to the unique challenges faced by healthcare systems in Latin America, ensuring accessibility and practical application.

Furthermore, companies specializing in surgical navigation technology should explore innovative models, such as leasing programs or collaborative partnerships, to make this technology more affordable and accessible worldwide. By lowering financial barriers and fostering awareness, we can ensure that the benefits of navigation systems in spine surgery reach all corners of the globe, ultimately advancing patient care standards everywhere.

## Appendices

Appendix A

**Table 4:** Study survey.

1. Sex:	2. You are:
a) Male.	a) Orthopedic surgeon
b) Female.	b) Neurosurgeon
	c) Orthopedic surgeon /Neurosurgeon with additional fellowship in spine surgery
3. Country	4. Name of the hospital
a) Mexico	
b) Chile	
c) Peru	
d) Argentina	
e) Brasil	
f) Guatemala	
g) Colombia	
h) El salvador	
i) Republica dominicana	
5. Are you involved in a spine surgery fellowship program in your hospital as a professor or as a fellow?	6. Are you an AO Spine member?
a) Yes, I'm a professor	a) Yes
b) Yes, I'm a fellow	b) No
c) No	
7. Years of experience in spine surgery?	8. How many surgeries do you do per year?
a) Less than 5	a) 0-50
b) 6-10	b) 51-100
c) 11-15	c) 101-150
d) 15-20	d) 151-200
e) >20	e) >200: Specify:
9. Distribution in percent of spinal pathologies that you routinely treat in your clinical practice.	10. How often does your technique need a fusion procedure?
Degenerative:	a) 0-25%
a) 0%	b) 26-50%
b) 1-25%	c) 51-75%
c) 26-50%	d) 76-100%
d) 51-75%	
e) 76-100%	
Tumor:	
a) 0%	
b) 1-25%	
c) 26-50%	
d) 51-75%	
e) 76-100%	
Infection:	
a) 0%	
b) 1-25%	
c) 26-50%	
d) 51-75%	
e) 76-100%	
Deformity:	
a) 0%	
b) 1-25%	
c) 26-50%	
d) 51-75%	
e) 76-100%	
Trauma:	
a) 0%	
b) 1-25%	
c) 26-50%	
d) 51-75%	
e) 76-100%	
11. According to your knowledge about computer-assisted navigation, would you use it?	12. Have you ever participated in a course of computer-assisted navigation surgery?
a) I don't have it, yes, I would use it.	a) Yes
b) I don’t have it; I wouldn’t use it.	b) No and I would like it.
c) Yes, I have, I already use it.	c) No and I wouldn't like it.
d) Yes, I have, I don’t use it.	
13. In percent, how many of your spine surgeries do you perform with:	14. What are the perceived advantages of navigation systems? (1: ¨essential¨, 2: ¨important¨ and 3: ¨unnecessary¨)
Computer-assisted navigation system?	a) Improves accuracy (level and position of the screw)
a) 0%	b) Decreases surgical time
b) 1-25%	c) Decreases radiation exposure for the surgical team
c) 26-50%	d) Ameliorates preoperative and intraoperative planning
d) 51-75%	e) Assists with complex (>5 levels, cervical)
e) 76-100%	f) Assists with revision surgeries
Free hand?	g) Provide support in case of medical-legal issues
a) 0%	h) Offers assistance in minimally invasive techniques
b) 1-25%	i) Intraoperative image control of screw position
c) 26-50%	
d) 51-75%	
e) 76-100%	
2D fluoroscopy?	
a) 0%	
b) 1-25%	
c) 26-50%	
d) 51-75%	
e) 76-100%	
15. What are the perceived disadvantages of navigation systems? (One or multiple options are possible)	16. If you chose the option "Increase the time of surgery," specify the time in minutes.
a) Not available	
b) It is expensive	
c) I do not know how to use it	
d) More radiation exposure	
e) It is not necessary	
f) Medical insurance reluctant to approve	
g) Incompatibility between systems available	
h) Increase the time of surgery	
